# Occurrence of Deoxynivalenol in Foods for Human Consumption from Tehran, Iran 

**Published:** 2014

**Authors:** Hassan Yazdanpanah, Alireza Shafaati, Seyed Mohsen Foroutan, Afshin Zarghi, Farshid Aboul-fathi, Arash Khoddam, Fatemeh Shaki, Firoozeh Nazari

**Affiliations:** a *School of Pharmacy, Shahid Beheshti University of Medical Sciences, Tehran, Iran.*; b *Food and Drug Organization, Iranian Ministry of Health and Medical Education, Tehran, Iran.*; c *Noor Research and Educational Institute, Tehran, Iran.*; d *Department of Pharmacology and Toxicology, School of Pharmacy, Mazandaran University of Medical Sciences, Sari, Iran. *

**Keywords:** Deoxynivalenol, HPLC, Dietary exposure, Monolithic column, Iran

## Abstract

The occurrence of deoxynivalenol (DON) in retail foods in Tehran (Iran) was determined using high-performance liquid chromatography technique and immunoaffinity column as the clean-up step. A method was validated for analysis of DON in rice, bread, puffed corn snack and wheat flour. The average recoveries and precision (RSD) for DON in different foods ranged 84.2-93.1% and 2.9-12.0%, respectively. A survey of DON was performed on the 72 samples of rice, bread, puffed corn snack, and wheat flour collected from Tehran retail market. The data showed that 10 samples (13.9%) out of 72 samples were contaminated with DON with the maximum level of 368.7 ng/g. The samples had contamination level lower than the maximum tolerated level of DON in foods in Iran. The total intake of DON was under the provisional maximum tolerable daily intake set for DON by the JECFA.

## Introduction

Mycotoxins are secondary metabolites produced by microfungi that are capable of causing disease and death in humans and other animals (1). Deoxynivalenol (DON) is one of the most important mycotoxins which are considered to be economically and toxicologically important in worldwide. It is produced by several *Fusarium *species, most commonly, *Fusarium graminearum *and *F. Culmorum *(2, 3). DON can accumulate in human and animal bodies and has teratogenic, neurotoxic, embryotoxic, immunosuppressive and acute effects (4-6). DON predominantly contaminates a number of cereals including wheat, corn, rye, rice and barley (7). The United States FDA has issued advisory levels of 1000 ng/g for wheat products for human consumption (8). FAO has issued advisory levels of 100–2,000 ng/g for DON in cereal and finished cereal products intended for human consumption (9). In Iran, maximum tolerated level (MTL) of DON in cereals including barley, maize, rice and wheat is 1,000 ng/g (10, 11). 

High performance liquid chromatography (HPLC) is the most widely used laboratory method for analysis of DON. Most reported methods are UV or fluorescent detection coupled with immunoaffinity column clean-up step and C_18_-reversed phase column (3, 4, 12 and 13). However, most of these methods are time-consuming or complex and therefore are not suitable for all conditions.

There is little data on the natural occurrence of DON in cereals and cereal products in Iran. In this study, we investigated the presence of DON in various foods collected from the Tehran retail markets using a rapid HPLC method. We also used the data to estimate DON intake by the population of Tehran.

## Experimental

*Equipment and reagents*

All reagents were of analytical grade. Solvents used for the experiments were of either HPLC or analytical grade. The standard of DON was purchased from Sigma-Aldrich as pure mycotoxins (MO, United States). The IAC for DON was purchased from Vicam Company, MA and USA. The chromatographic apparatus consisted of a model Wellchrom K-1001 pump, a model Rheodyne 7125 injector and a model K 2501 UV detector connected to a model Eurochrom 2000 integrator, all from Knauer (Berlin, Germany). The separation was performed on Chromolith Performance (RP-18e, 100 × 4.6 mm) column from Merck (Darmstadt, Germany). Glass microfiber filters (pore size: 1 μm and/or 1.5 μm) were purchased from Vicam Company, MA, USA. 

*Sampling and sample preparation*

Seventy two samples including 18 rice, 18 puffed corn snack, 18 wheat flour and 18 “lavash” bread samples were collected by a trained person from various sales outlets in nine geographical zones in Tehran, Iran in June 2005 according to the sampling method for the official control of the levels for certain contaminants in foodstuffs (14). About 1-5 kilograms of the samples were collected, labeled, packaged and taken to the laboratory. The both “lavash” bread samples and wheat flour samples (used for preparation of “lavash” bread samples) were collected from “lavash” bread bakeries. Regarding puffed corn snack, the packaged samples (each 65 g) were collected from retail market. The samples were finely ground by mill and/or blender, mixed thoroughly and subsamples stored in freezer at -32º C until analysis.

*DON analysis*

After preparation of stock standard solution of DON, the concentration was determined using UV spectrophotometer. This standard was used to prepare mixed working standards for HPLC analysis. Samples were analyzed using a HPLC method with some modification (4). Twenty five grams of bread and rice samples were extracted with water. Samples of puffed corn snack and wheat flour were extracted with acetonitrile (60%). After filtration, the extract was diluted with water and filtered through glass microfiber filter. For cleanup of samples, DONtest IACs were used. Accordingly, 1 mL of the filtrate was passed through the IAC. The column was washed twice with 5 mL deionized water and dried under vacuum. Finally, DON was eluted from IAC with 1 mL methanol. The eluate was dried and then reconstitute with 300 μL acetonitrile-water (10:90, v/v) and then 100 μL was injected into the HPLC. Mobile phase was acetonitrile-water (10:90, v/v) with a flow rate of 1 mL/min. The UV detector was operated at 218 nm. 

*Method validation*

Recovery experiments were performed for determination of accuracy and precision of the method. Each test was performed three times and the mean recovery values are shown in Table 1. The values for percent recovery of DON from spiked rice, bread, puffed corn snack and wheat flour (RSD as % are given in parentheses) were 84.16% (12.00%), 84.96% (2.88%), 93.10% (11.32%) and 92.10% (8.27%), respectively (Table 1). These values fall well within EU method performance criteria for DON analysis (15). Calibration curves were obtained by six standards at the range of 0.1-5 μg/mL. The regression coefficient (*r*2>0.997) obtained indicated a good linearity of the analytical response. The limit of detection (LOD), signal-to-noise ratio of 3:1, and limit of quantification (LOQ), signal-to-noise ratio (s/n) of 9:1 were 2 and 6 ng/g, respectively.

**Table 1:** Results of validation assessment of HPLC method developed for determination of DON in different foods (n=3).

**Sample**	**Spiking level**	**Recovery %**	**RSD**_r_
Rice	500	95.5	5.5
	1000	76.2	3.2
	2000	80. 8	14.0
	Mean recovery ± SD	84.16±10.10	12
Bread	500	82.5	15.8
	1000	87.4	5.3
	2000	85	11.8
	Mean recovery ± SD	84.96±2.45	2.88
Puffed corn snack	500	105.1	11.4
	1000	88.9	13.7
	2000	85.3	6.8
	Mean recovery ± SD	93.10±10.54	11.32
Wheat flour	500	82.7	8.9
	1000	96.68	9.9
	2000	96.9	1.8
	Mean recovery ± SD	92.10±8.13	8.27

**Figure 1:**
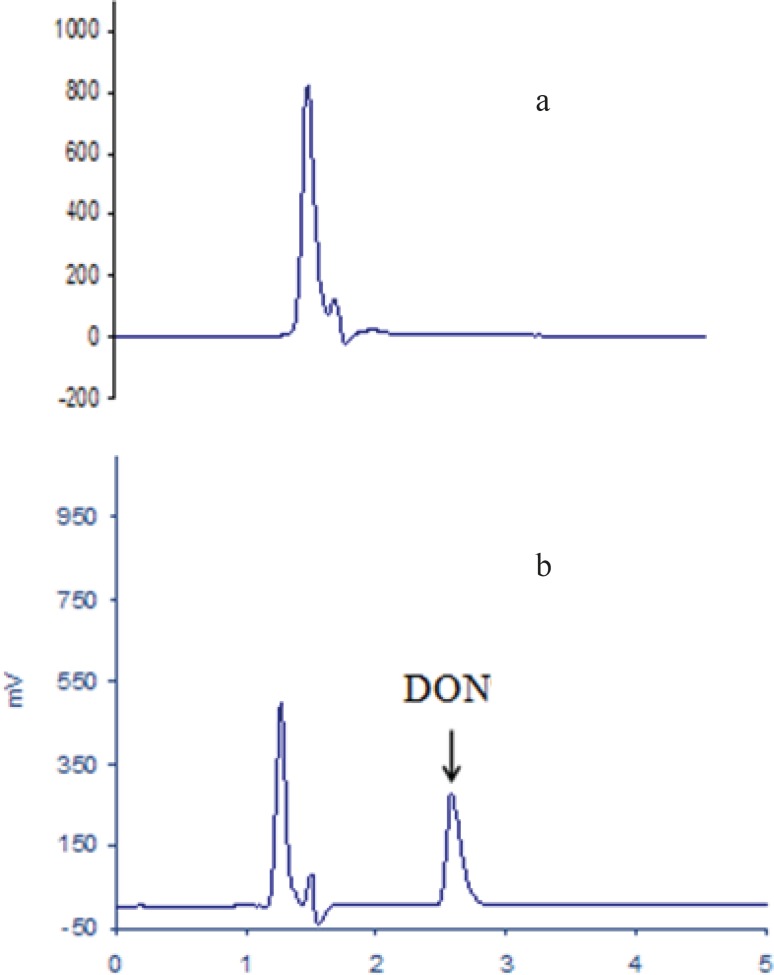
HPLC chromatograms of a) blank bread, and b) DON standard (1000 ng/mL).

The IAC applied for purification of this toxin eliminated false positive result and cleared peak without interfering compound obtained. In our study, due to using a short monolithic column, the total run time decreased to about 3 min. Typical chromatograms for DON are shown in Figure 1. This method is well suited for routine analysis of DON.

*Quality assurance*

To evaluate the reliability of the results, in addition to applying regular validation assessment to the developed method, internal quality control experiments were also performed. In each working day, a blank and a spiked sample were analyzed. According to the recovery values, DON levels were corrected for recoveries. In addition, a certified reference material (CRM) from FAPAS (UK) was analyzed.

*Exposure assessment of DON*

Exposure to mycotoxins for each type of food depends on the mycotoxins concentration in food and the amount of food consumed. The rates of rice and bread consumption were based on a consumption survey performed in Iran since 2001-2003 (16). Average consumptions of rice and bread in adults are 107 g and 286 g per day per person in Tehran, respectively (16). There are no official data of puffed corn snack consumption in Iran. So, we assumed mean of consumption is a package (65 g) per day.

For estimation of DON dietary intake, deterministic methods were performed combining food daily intake (per body weight) with mean concentration of DON in food as follows: individual DON exposure (μg/Kg bw/day) = (daily food intake/body weight) × (mean concentration of DON in food).

## Result and Discussion

*Occurrence of DON in retail foods*

The data showed that the level of DON in 62 out of 72 samples was lower than the LOQ (6 ng/g) and in 10 samples, level of DON contamination was between 6.0 ng/g and 368.7 ng/g. DON was detected only in one sample of rice and bread. Range of contamination in 8 positive puffed corn snack samples was between 60.2 and 368.7 ng/g (Table 2).

**Table 2:** Contamination data for DON (ng/g) in rice, bread, puffed corn snack and wheat flour samples marketed in Tehran, Iran

**Sample **	**NO. of samples **	**Sample positive (%) **	**Mean(±SD)**a	**Median **	**Max **
Rice	18	1 (5.6 )	345.0	-	345.0
Bread	18	1 (5.6 )	120.5	-	120.5
Puffed corn snack	18	8 (44.4)	113.2 (104±)	74.0	368.7
Wheat flour	18	0	-	-	-

^a^Mean of positive samples

There is only one report on DON contamination in Iran. In 2004-2005, Karimi-Osboo determined DON in corn samples produced in Golestan and Ardabil Provinces, Iran, and found DON in 76.7% of samples in the range of 54.4-518.4 ng/g (17). The mean of contamination was 116.25 ng/g. In Turkey, DON was detected in six out of 68 cereal samples and in none of 15 pulse products collected from markets and street Bazaars (18). The maximum detected level of DON was 2.67 mg/Kg in a corn flour sample. In a 3-year survey in South Korea, the concentration of DON was determined in cereal-based foods (19). Among 689 samples, 272 samples (39%) were contaminated with DON. Relatively high DON levels were detected in dried corn, with a mean concentration of 109 μg/Kg. González-Osnaya *et al. *found DON in 28.0% and 62.6% of the bread and pasta samples, with mean content of 42.5 and 137.1 μg/Kg, respectively (20). In South Africa, 69.6 % of wheat flour samples were contaminated with DON at the levels equal to or below 100 μg/Kg (mean of 16 positives, 29 μg/Kg) (21). 

*Exposure assessment of Tehran population to DON*

There are some published papers regarding exposure assessment of Tehran population to AFB1 and zearalenone (22- 23). In this study, the estimated daily intake of DON was studied and is shown in Table 3. The mean dietary exposure of DON through all food products consumption was less than the PMTDI of 1 μg/Kg bw/day (24) (Table 3*)*. 

**Table 3:** Estimated daily intake of DON (ng/Kg bw/day) through consumption of rice, bread and puffed corn snack marketed in Tehran, Iran

**Samples**	**Mean**a,b (ng/g)	**Mean Daily intake(ng/kg bw/day)**a,c
Rice	22.00	33.63
Bread	9.53	38.92
Puffed corn snack	48.97	45.48

a: Mean of all samples.

b: The not detected values were replaced by half the limit of quantification.

c: Body weight (bw) for adults is assumed 70 Kg.

In South Korea, daily intake of DON was estimated to be between 0.066 and 0.142 μg/Kg bw for males and between 0.066 and 0.144 μg/ Kg bw for females. The young children (3–6 yr) showed the highest relative intake, with a mean intake of 0.142 μg/Kg bw/d for males and 0.144 μg/Kg bw/d for females (19). Soubra *et al*. (2009) calculated DON intakes for average and high consumers (75th and 95th percentile) among children and teenagers in Beirut (Lebanon). The intakes of DON were found to be below the PMTDI of 1 μg/Kg bw/day (25). In Japan, the intake of DON was estimated based on its presence in wheat using a probabilistic computer simulation method. The results showed that children aged 1-6 years old had the highest DON intake. The 99th percentile of simulated DON intake in the 1-6 years old group was greater than PMTDI (26). 

In this study, the specifically developed HPLC method was found to be accurate, precise and rapid and meets EU method performance criteria for DON analysis. Upon applying the proposed method, none of the various food samples had contamination more than the Iranian MTL. The total intake of DON was under the PMTDI set for DON by the JECFA. 
